# Erratum to: Microcephalic Osteodysplastic Primordial Dwarfism, Type II: a Clinical Review

**DOI:** 10.1007/s11914-017-0389-5

**Published:** 2017-07-15

**Authors:** Michael B. Bober, Andrew P. Jackson

**Affiliations:** 1 Stanley Kimmel Medical College, Thomas Jefferson University, Philadelphia, PA USA; 2 A. I. DuPont Hospital for Children, 1600 Rockland-Road, Wilmington, DE 19803 USA; 3 MRC Human Genetics Unit, Institute of Genetics and Molecular Medicine, University of Edinburgh, Edinburgh, EH4 2XU UK

**Erratum to: Curr Osteoporos Rep** (**2017) 15(2):61–69**

**DOI** 10.1007/s11914-017-0348-1

The article Microcephalic Osteodysplastic Primordial Dwarfism, Type II: a Clinical Review, written by Michael B. Bober and Andrew P. Jackson, was originally published Online First without open access. After publication in volume 15, issue 2, page 61–69 the author decided to opt for Open Choice and to make the article an open access publication. Therefore, the copyright of the article has been changed to © The Author(s) 2017 and the article is forthwith distributed under the terms of the Creative Commons Attribution 4.0 International License (http://creativecommons.org/licenses/by/4.0/), which permits use, duplication, adaptation, distribution and reproduction in any medium or format, as long as you give appropriate credit to the original author(s) and the source, provide a link to the Creative Commons license, and indicate if changes were made.

